# Role of Multidetector Computed Tomography in the Evaluation of Maxillofacial Trauma

**DOI:** 10.7759/cureus.35008

**Published:** 2023-02-15

**Authors:** Buchipudi Sandeep Reddy, Deepti Naik, Deepika Kenkere

**Affiliations:** 1 Radio-Diagnosis, Sri Devaraj Urs Academy of Higher Education and Research, Kolar, IND; 2 Oral and Maxillofacial Surgery, Sri Devaraj Urs Academy of Higher Education and Research, Kolar, IND

**Keywords:** fractures of nasal bone, mandibular bone fracture, maxillo-facial trauma, dental radiology, dentistry, sagittal, coronal, axial, multislice computed tomography, maxillo-facial fractures

## Abstract

Introduction: Maxillofacial fractures are among the commonest injuries occurring in trauma patients. Multislice computed tomography (CT) is a widely used radiological investigation that accurately reveals the number, location, and extent of the fractures as well as concomitant soft tissue injuries and has been found to be superior in the diagnosis of maxillofacial fractures owing to high sensitivity and specificity. This study was performed to assess the efficacy of axial, coronal, sagittal, and three-dimensional (3D) reformatted images in the detection of fractures in maxillofacial trauma. Materials and Methods: This was a cross-sectional descriptive study conducted on 49 adult patients with maxillofacial injuries undergoing multislice CT using a multidetector SiemensSOMATOM Emotion eco 16 slice CT scanner (Siemens AG, Munich, Germany). CT protocol consisted of non-contrast axial 16-slice helical series beam collimation ~ 3 mm, pitch ~ 0.8 - 1, tube current ~ 270 mAs, voltage ~ 130 kV, Total exposure time ~ 18 seconds, total radiation ~ 200 mGy. Along with the axial, coronal and sagittal images were reconstructed with 0.5 mm increment. 3D volume-rendering images were also obtained. 3D images were compared with axial images, coronal and sagittal plane images.

Results: The maximum number of cases was in the age group of 21-30 years with the male: female ratio being 5.12:1. The most common cause of injury was road traffic accidents (RTA). Mandible fractures were found to be the most common (20 patients, 40.8%) followed by fractures of nasal bone (18 patients, 36.7%). The incidence of frontal bone fractures was found to be the least (six patients, 12.24%). Our study found that 3D images are superior to axial in assessing the extent and degree of displacement of maxillofacial fractures in general. The maxillary sinus was found to be the most commonly fractured sinus (19 patients, 38.7%). Sphenoid sinus fractures were the least common (seen in two patients, 4.08%). CT findings correlated with the operative findings in most types of fractures. Conclusion: Multidetector CT with multiplanar and 3D reformation is highly accurate in the identification of fractures and assessing the extent and degree of displacement of fractures; hence, it is the imaging modality of choice in maxillofacial trauma. 3D images are much better for the detection of maxillofacial fractures compared to axial, coronal, or sagittal views, especially in maxilla and mandibular bone fractures. It is also found to be better at providing information on the patterns of the fracture lines and the displacement of the fracture fragments. Another added advantage of multidetector CT is that it is a non-invasive technique with good accuracy and a short scan time.

## Introduction

Maxillofacial fractures are among the most common injuries occurring in trauma patients seen along with varying degrees of physical, structural, and aesthetic damage [1]. Facial bones that are highly susceptible to trauma include the mandible, maxilla, zygomatic arch, nasal bones, and orbit; all of which lie in close proximity with the cranial structures making untreated fractures a possible trigger for infections, serious complications and, in some cases, even death [2,3]. Multislice computed tomography (CT) is a widely used radiological investigation that accurately reveals the number, location, and extent of the fractures as well as concomitant soft tissue injuries. CT is thus gradually replacing the conventional radiograph (Waters view and axial films) for maxillofacial trauma and is increasingly being performed for the evaluation and classification of facial trauma [4]. Previously, two-dimensional CT images had a limitation as a diagnostic tool in complicated maxillofacial fractures. However modern imaging modalities have evolved to include three-dimensional (3D) CT, which has greater value in the evaluation and management of acute facial trauma. Surgeons frequently need to make their own evaluation of the degree of skeletal disruption and this is excellently revealed by imaging studies when planning the fixation of facial fractures [5]. Multidimensional CT has been found to be superior in the diagnosis of maxillofacial fractures owing to high sensitivity and specificity [6]. This study was performed to assess the efficacy of axial, coronal, sagittal, and 3D reformatted images in the detection of fractures in maxillofacial trauma.

## Materials and methods

This was a cross-sectional descriptive study conducted at a tertiary hospital, R.L. Jalappa Hospital And Research Centre in Kolar, Karnataka, India, over a period of 18 months (January 2021 to June 2022). All adult patients with maxillofacial injuries undergoing multislice CT examination who were shown to be positive for fractures were included in the study. Images with motion and other types of artifacts on CT scans, patients with past history of maxillofacial surgery or intervention, or facial deformities or tumors like exostosis and fibrous dysplasia were excluded from the study.

The sample size was calculated as 43 using OpenEpi software version 3.01 [7]. The study was approved by the Institutional Ethics Committee of Sri Devaraj Urs Medical College, Kolar, Karnataka, India (Approval number: SDUMC/KLR/IEC/600/2020-21). Informed written consent was obtained from all the study participants, and only those participants willing to sign the informed consent were included in the study. The risks and benefits involved in the study and the voluntary nature of participation were explained to the participants before obtaining consent. The confidentiality of the study participants was maintained. 

Patients satisfying the inclusion criteria underwent the CT evaluation after giving consent. All the CT scans in this study were performed using a multidetector Siemens SOMATOM Emotion eco 16-slice CT scanner (Siemens AG, Munich, Germany). CT protocol consisted of non-contrast axial 16-slice helical series beam collimation ~ 3 mm, pitch ~ 0.8 - 1, tube current ~ 270 mAs (milliampere-seconds), voltage ~ 130 kV (kilovolt), total exposure time ~ 18 seconds, total radiation ~ 200 mGy (milligray). Along with the axial, coronal and sagittal images were reconstructed with 0.5 mm increment. 3D volume-rendering images were also obtained. 3D images were compared with axial images and coronal and sagittal plane images. The interpretation of CT was done by the principal investigator with the assistance of an experienced clinician. Other demographic data such as age, sex, and cause were also documented in a structured questionnaire to know the prevalence of maxillofacial injuries. Data were entered into a Microsoft Excel sheet (Microsoft Corporation, Redmond, Washington, United States), and IBM SPSS Statistics for Windows, Version 20.0 (Released 2011; IBM Corp., Armonk, New York, United States) was used for statistical analysis.

## Results

A total of 49 subjects were included in the study of which the maximum number of cases were in the age group of 21-30 years (21 patients; 42.8%), followed by 12 (24.4%) in the 31-40 years age group, eight (16.3%) in 18-20 years age group, six (12.2%) in the 41-50 years age group, and two (4%) in the 51-60 years age group. The mean age group of subjects was 31.04+/-10.9 years. The age group of 51-60 years (two patients, 4%) was found to be the least affected. This is shown in Table 1.

**Table 1:** Distribution of subjects based on age

Age (years)	Number (n)		Total	%
	Male	Female		
18-20	8	0	8	16.3
21-30	18	3	21	42.8
31-40	7	5	12	24.4
41-50	6	0	6	12.2
51-60	2	0	2	4
Total	41	8	49	100

A total of 41 (84%) patients were male and formed the majority of the study population, while eight (16%) patients were female. The male:female ratio was 5.12:1. The most common cause of injury was road traffic accidents (95.9%) followed by falls from height and assault (2.04% each).

**Figure 1:**
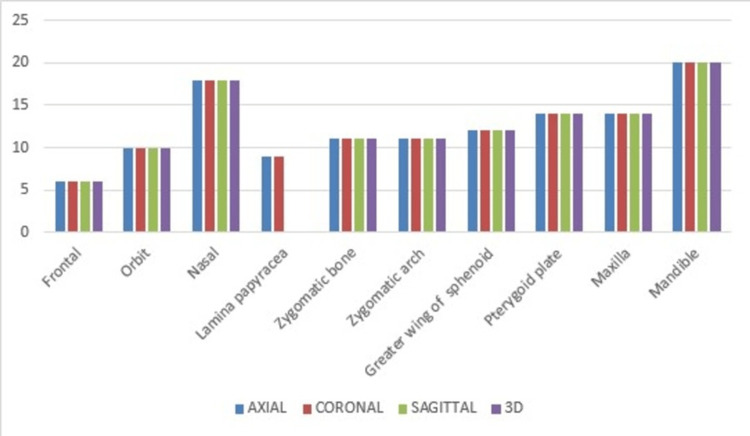
Incidence of bone fracture detection in various views

Mandible fractures were found to be the most common (20 patients, 40.8%) followed by fracture of nasal bone (18 patients, 36.7%), pterygoid plate and maxilla fractures (14 patients, 28.6%), greater wing of sphenoid (12 patients, 24.4%), zygomatic bone and arch (11 patients each, 22.4%), orbit fractures (10 patients, 20.4%), and lamina papyraceae fractures (nine patients, 18.36%). Incidence of frontal bone fractures was found to be the least (six patients, 12.24%) The incidence of bone fracture detection in various views are shown in Figure 1.

Incidence of sinus fractures

**Figure 2:**
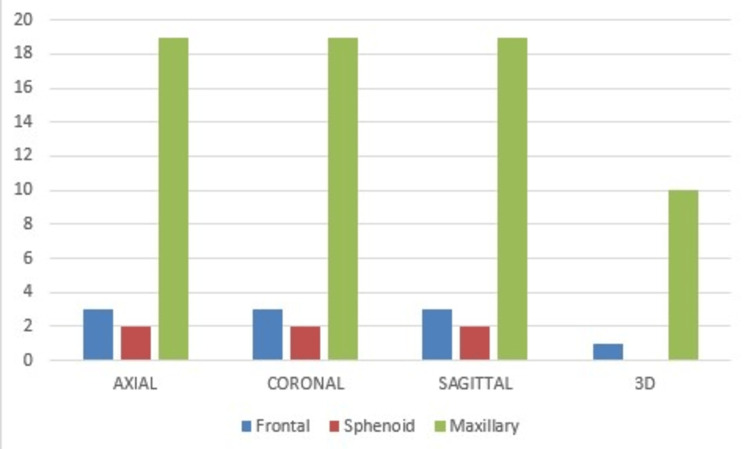
Incidence of sinus fractures in various views.

**Figure 3:**
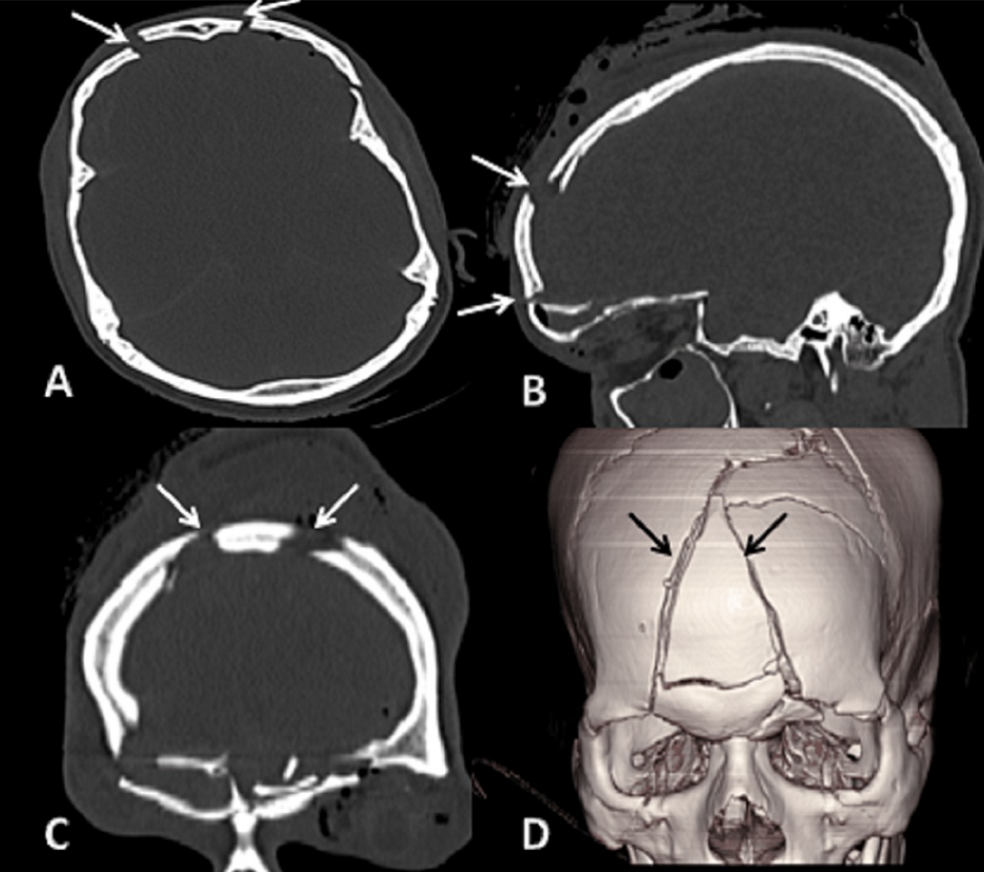
CT scan: (A) axial, (B) sagittal, (C) coronal sections bone window, and (D) 3D reformatted image showing fracture of the bilateral frontal bones. 3D: three dimensional

**Figure 4:**
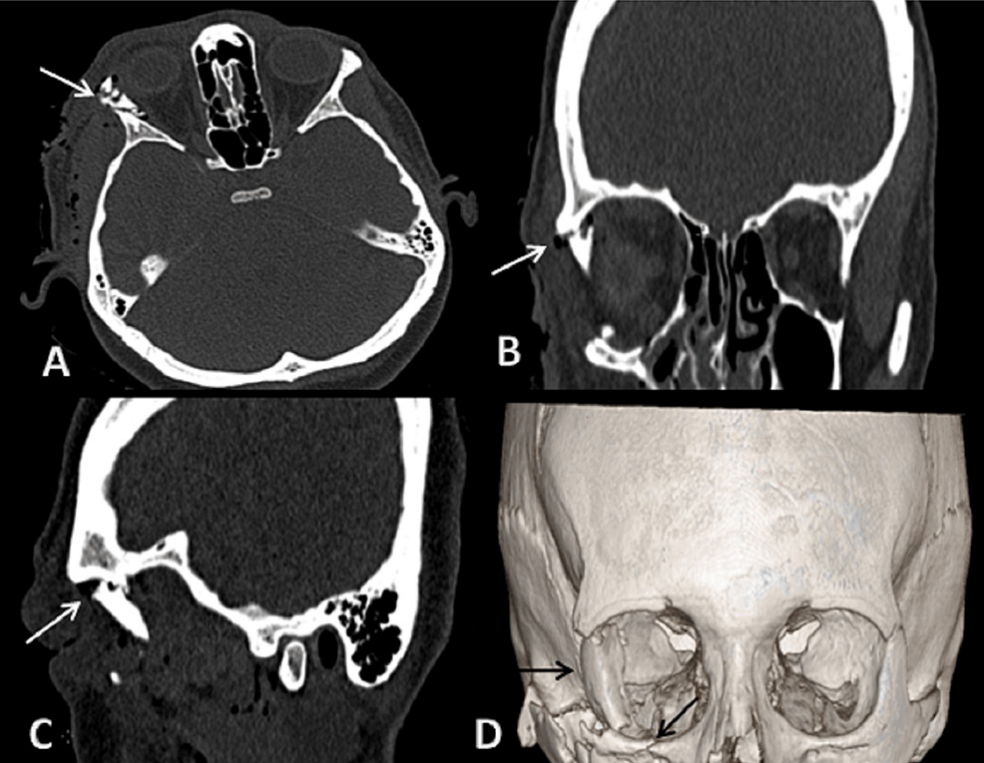
CT scan: (A) axial, (B) coronal, (C) sagittal sections bone window, and (D) 3D reformatted image showing fracture of lateral wall of right orbit. On 3D reformatted image, we can also appreciate orbital floor fracture and displacement of fracture fragments. 3D: three dimensional

**Figure 5:**
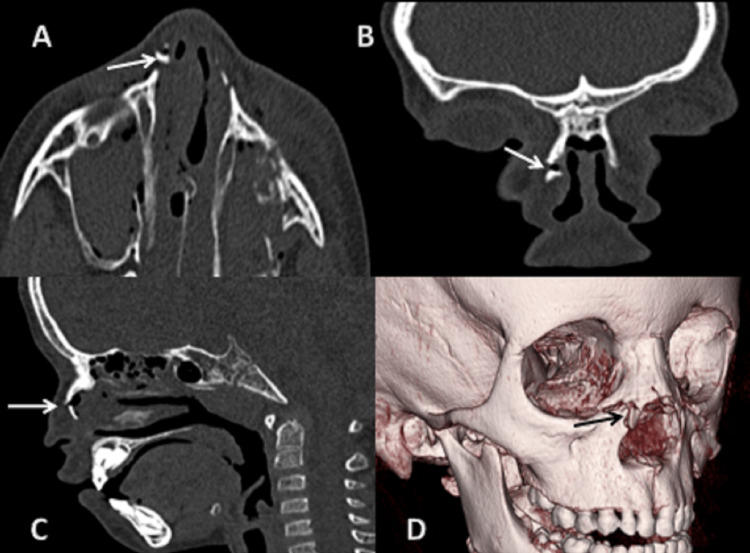
CT scan: (A) axial, (B) coronal, (C) sagittal sections bone window, and (D) 3D reformatted image showing fracture of right nasal bone with displacement of fracture fragment. 3D: three dimensional

**Figure 6:**
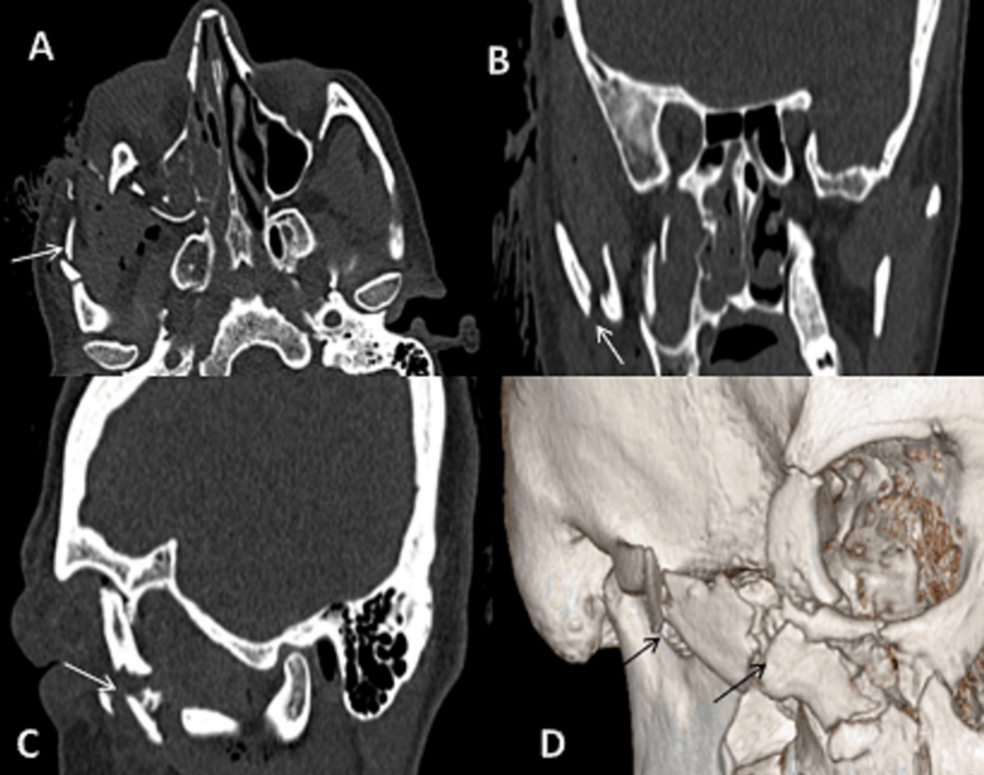
CT scan: (A) axial, (B) coronal, (C) sagittal sections bone window, and (D) 3D reformatted image showing comminuted fracture of zygomatic bone and zygomatic arch on right side with displacement of fracture fragments. 3D: three dimensional

**Figure 7:**
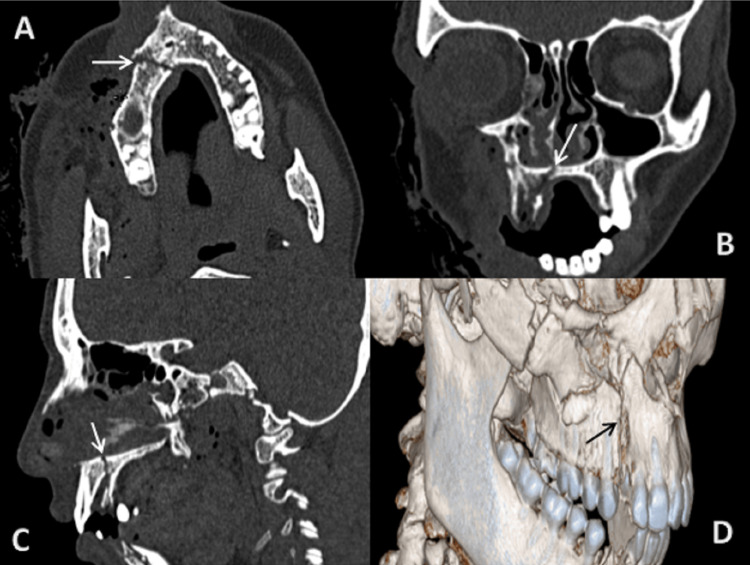
CT scan: (A) axial, (B) coronal, (C) sagittal sections bone window, and (D) 3D reformatted image showing fracture of alveolar process of maxilla extending to hard palate on right side. 3D: three dimensional

**Figure 8:**
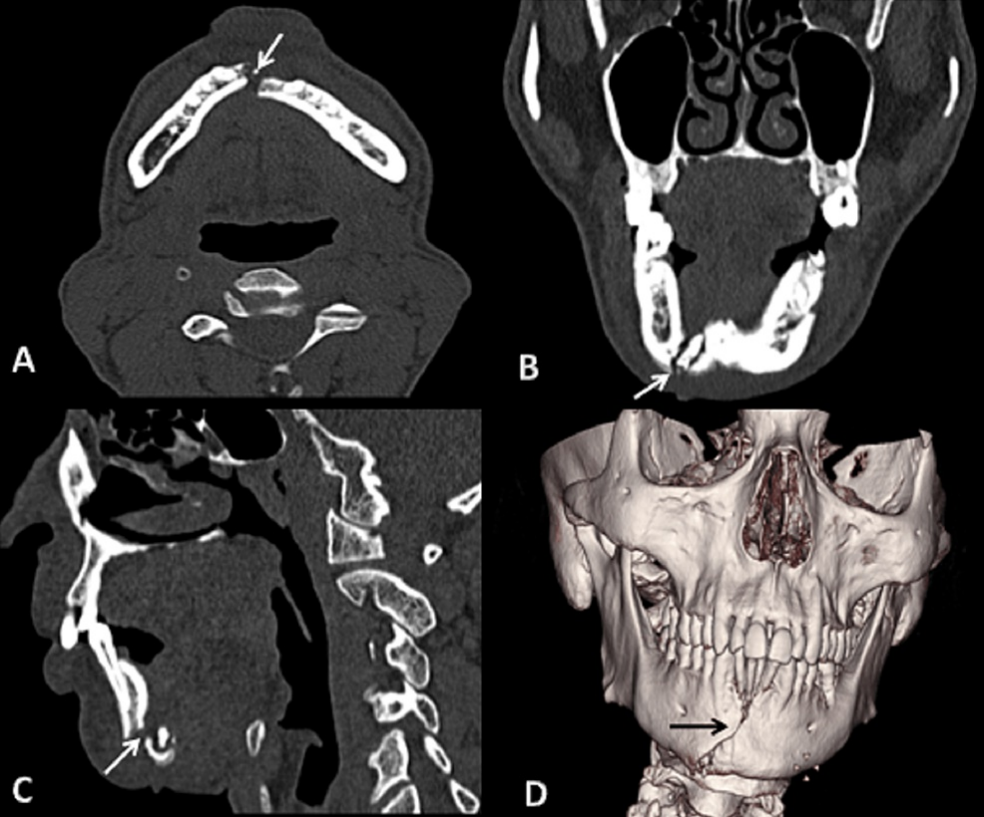
CT scan: (A) axial, (B) coronal, (C) sagittal sections bone window, and (D) 3D reformatted image showing fracture of symphysis menti of mandible extending to parasymphysis on right side. 3D: three dimensional

**Figure 9:**
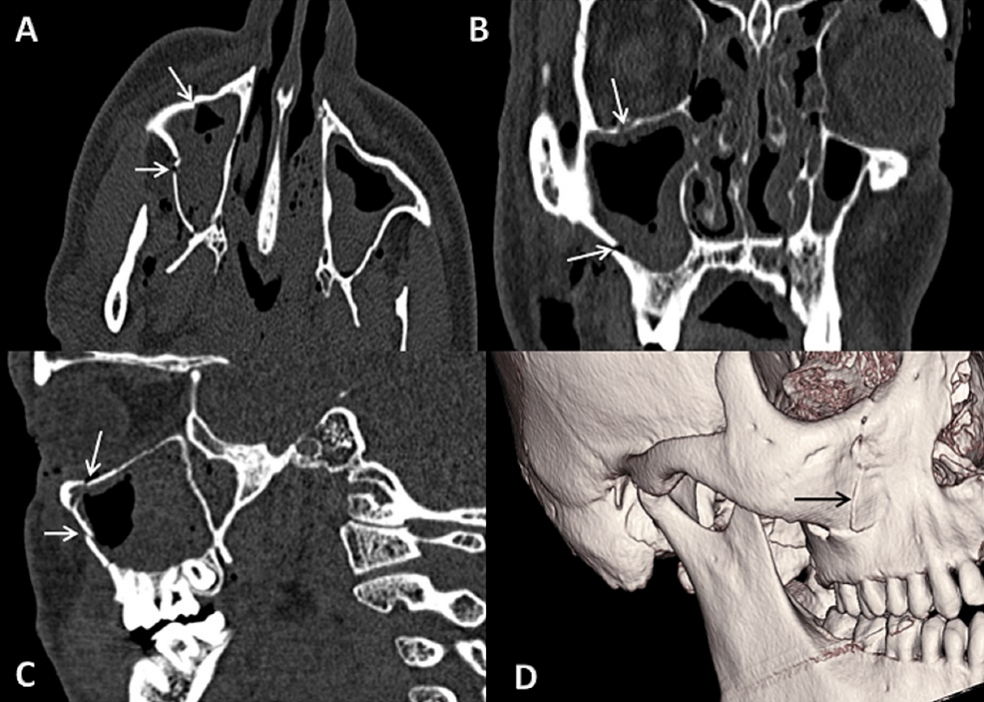
CT scan: (A) axial, (B) coronal, (C) sagittal sections bone window, and (D) 3D reformatted image showing fracture of anterior and postero-lateral walls of the right maxillary sinus. 3D: three dimensional

Maxillary sinus was found to be the most commonly fractured sinus (19 patients, 38.7%) as per the findings of our study, Frontal sinus fractures were seen in three patients (6.1%), and sphenoid sinus fractures were the least common (seen in two patients, 4.08%). Figure 2 shows the incidence of various sinus fractures noted in patients with maxillofacial fractures in our study. Almost all facial bone fractures are assessed in axial, coronal, sagittal, and 3D reformatted images to detect fractures in maxillofacial trauma. Study case images are shown in Figures 3-9.

## Discussion

Multidetector CT provides views in three different planes along with high-quality 3D reconstruction which provides excellent visualization and helps in the interpretation of the fracture segments whether comminuted or displaced, even in the case of complex fractures. This makes it easier for maxillofacial surgeons to plan surgeries efficiently [8]. In the present study, the maximum number of patients (43%) were in the age group of 21-30 years of which 84% were males and constituted the majority. The male: female ratio was 5.12:1 and the mean age group was found to be 31.04+/-10.9 years. These findings are in agreement with similar studies by Tomich et al., Chandra Shekar BR et al, and Subhashraj et al., where most patients were in the age group of 21-30 years with a male preponderance, and road traffic accidents were the most common etiology for maxillofacial trauma [5,9,10]. In the study by Tomich et al., maxillofacial trauma was common in the 15-35 years age range [5].

In our study, the most common cause of injury was road traffic accidents (95.9%) followed by falls from height and assaults (2.04% each). Similarly, it has been noticed by other authors such as Singh et al. that the incidence of maxillofacial fractures is continually on the rise due to uncontrolled traffic and overpopulation resulting in road traffic accidents being the main etiological element in maxillofacial fractures as noted in their study as well [11].

In our study, we found that almost all facial bone fractures are accurately assessed in axial, coronal, sagittal, and 3D reformatted images to detect fractures in maxillofacial trauma. Mandible fractures were found to be the most common and frontal bone fractures were found to be the least. All four views (axial, coronal, sagittal, and 3D) were equally useful in detecting these fractures.

In the study by Subhashraj et al., mandibular fractures were most common and fractures of the zygomaticomaxillary region followed closely [10]. Hwang et al. also conducted a study on patients who sustained maxillofacial fractures but found the nasal bone to be the most affected [12]. Zygomaticomaxillary fractures were also quite common in their study (14%); whereas in our study, nasal bone fractures were the second-most common. Similarly, Patil et al. also found the nasal bone to be the most common bone to be fractured, followed closely by naso-ethmoidal and zygomatic-maxillary complex fractures [13]. Axial and coronal images were found to be better for fractures of orbit fractures. All four views (axial, coronal, sagittal as well as 3D) were equally useful in the detection of nasal, zygomatic bone fractures, and fractures of the greater wing of sphenoid. We found that lamina papyracea fractures are not assessed in sagittal and 3D images and are seen best in axial and coronal images. 3D and axial images were better than axial and coronal sections for detecting fractures of the zygomatic arch. Axial and coronal images are better for fractures of pterygoid plates compared to sagittal and 3D images. 3D images are much better for detecting maxilla and mandible fractures than axial, coronal, or sagittal views. In addition, our study found that 3D images are superior to axial in assessing the extent and degree of displacement of maxillofacial fractures in general. 

Among the sinuses, maxillary sinus was found to be the most commonly fractured sinus (19 patients, 38.7%) in our study, frontal sinus fractures were seen in three patients (6.1%), and sphenoid sinus fractures were the least common (seen in two patients, 4.08%). Paranasal sinuses could not be assessed in 3D images. Maxillary sinus fractures were easily detected on axial, coronal, and sagittal views. However, it was observed that medial wall fractures cannot be well visualized in 3D; hence, only 10 out of 19 cases were detected on 3D images. On the contrary, it was noted that anterior and posterolateral wall fractures were better visualized in 3D. Communited displaced fractures were also better visualized in 3D.

Frontal sinus fractures were seen in three patients (6.1%), with only two (4.08%) being detected on all four views (axial, coronal, sagittal as well as 3D). Posterior wall fractures were not well visualized in 3D, but no such difficulty was encountered for anterior wall fractures. Communited displaced fractures were also visualized in 3D. These findings are similar to the findings of Fox et al., who reported that 3D images in multidetector CT scans are rapid and provide a better view thereby aiding the detection of maxillofacial fractures, especially those of the zygomatic bone [14]. They also found that medial wall fractures in the case of maxillary fractures were seen better on axial CT, but there was no difference between the medial and lateral maxillary sinus fractures. They also found axial scans to be superior to coronal in detecting zygomatic arch fractures. This is another finding that has been substantiated by an author of a similar study, Tanrikulu et al., who concluded that axial CT is best for zygomatic arch fractures with no difference between axial and coronal images [15].

Our study found that the detection of maxillofacial fractures, especially displaced and communited ones can be detected ideally by 3D images. Bernard et al. seem to be in agreement with this finding, according to whom, coronal CT is the best method when compared to axial CT in the detection of mandibular fractures [16]. Fox et al. further mentioned their experience with 3D CT scans and found them to be less reliable than axial images in the detection of orbital fractures in the detection, extent, and displacement of fractures when compared with axial and coronal images [14]. CT findings were found to be correlating with the operative findings in most types of fractures.

## Conclusions

Multidetector CT with multiplanar and 3D reformation is highly accurate in the identification of fractures and assessing the extent and degree of displacement of fractures; hence, it is the imaging modality of choice in maxillofacial trauma. 3D images are much better for the detection of maxillofacial fractures compared to axial, coronal, or sagittal views, especially maxilla and mandible fractures. It is also found to be better at providing information on the patterns of the fracture lines and the displacement of the fracture fragments. Another added advantage of multidetector CT is that it is a non-invasive technique with good accuracy and a short scan time.
